# Modulation of the BRCA1 Protein and Induction of Apoptosis in Triple Negative Breast Cancer Cell Lines by the Polyphenolic Compound Curcumin

**DOI:** 10.4137/bcbcr.s3067

**Published:** 2009-09-02

**Authors:** Danica L. Rowe, Tuba Ozbay, Ruth M. O’Regan, Rita Nahta

**Affiliations:** 1 Departments of Pharmacology; 2 Hematology and Medical Oncology; 3 Winship Cancer Institute; 4 Molecular and Systems Pharmacology Program, Graduate Division of Biological and Biomedical Sciences, Emory University School of Medicine, Atlanta, GA 30322

**Keywords:** mammary carcinoma, triple negative, curcumin, DNA damage, BRCA1

## Abstract

In the current study, we sought to examine the effects of curcumin in a specific type of breast cancer called triple negative breast cancer. These cancers lack expression of the estrogen and progesterone receptors and do not over-express HER2. Current treatment for triple negative breast cancers is limited to cytotoxic chemotherapy, and upon relapse, there are not any therapies currently available. We demonstrate here that the bioactive food compound curcumin induces DNA damage in triple negative breast cancer cells in association with phosphorylation, increased expression, and cytoplasmic retention of the BRCA1 protein. In addition, curcumin promotes apoptosis and prevents anchorage-independent growth and migration of triple negative breast cancer cells. Apoptosis and BRCA1 modulation were not observed in non-transformed mammary epithelial cells, suggesting curcumin may have limited non-specific toxicity. This study suggests that curcumin and potentially curcumin analogues should be tested further in the context of triple negative breast cancer. These results are novel, having never been previously reported, and suggest that curcumin could provide a novel, non-toxic therapy, which could lead to improved survival for patients with triple negative breast cancer. Curcumin should be studied further in this subset of breast cancer patients, for whom treatment options are severely limited.

## Introduction

Curcumin (diferuloyl methane) is a natural yellow-pigmented polyphenol component of the spice turmeric, which is derived from the roots of the *Curcuma longa* plant indigenous to Southeast Asia. Curcumin has been used as an anti-inflammatory agent in traditional Indian Ayurvedic medicine for centuries.^1^ Anti-tumor effects of curcumin have been reported in numerous pre-clinical models of solid tumors including pancreatic, colorectal, prostate, and breast.^2^–^7^ In breast cancer cell lines, curcumin activated cell cycle arrest and apoptosis by inhibiting cyclin-dependent kinase (cdk) activity, suppressing cyclin D1 and cyclin E expression, increasing levels of cdk inhibitors p21 and p27, and inducing p53 transcriptional activity.^8^–^10^ Many of the molecular effects of curcumin have been attributed to its ability to potently inhibit transcriptional activity of nuclear factor kappa B (NF-kB), leading to reduced expression of anti-apoptotic, proliferative, pro-angiogenic, and metastatic target genes of NF-kB, with subsequent inhibition of mammary tumorigenesis and metastasis *in vivo*.^7^ In addition, signaling from the epidermal growth factor receptor (EGFR) and human epidermal growth factor receptor 2 (HER2) is suppressed in EGFR- or HER2-over-expressing breast cancer cells, with reduced downstream ERK1/2, JNK, and Akt activity.^11^,^12^ Importantly, apoptosis in response to curcumin appears to be far more pronounced in cancer cell lines versus non-tumorigenic breast epithelial cells.^12^,^13^

In the current study, we sought to examine the effects of curcumin in breast cancer cells that lack expression of estrogen receptor (ER) and progesterone receptor (PR), and do not over-express HER2, thus, conferring so-called triple negative expression status.^14^ Triple negative breast cancers (TNBCs) occur more frequently in pre-menopausal females of African-American and Hispanic descent, and display very aggressive behavior with shorter post-relapse survival relative to other breast cancer types.^15^–^17^ Due to the absence of ER and lack of HER2 over-expression, TNBCs are not treated with endocrine or HER2-targeted therapies. Instead, standard first-line treatment for patients with TNBC is cytotoxic chemotherapy.

Common molecular changes observed in TNBC include *p53* mutation, EGFR over-expression, and dysfunction in the BRCA1 pathway.^14^ The BRCA1 tumor suppressor protein is a critical mediator of DNA repair in response to double-strand breaks.^18^–^21^ Breast cancers with BRCA1 dysfunction show a high frequency of chromosomal abnormalities.^22^–^24^ In addition, since BRCA1 mediates repair of DNA strand breaks, loss of BRCA1 makes cancer cells more susceptible to apoptosis after treatment with DNA damaging drugs such as anthracyclines and platinum agents.^25^

Patients with TNBC who achieve pathologic complete response (pCR) to chemotherapy tend to have a good prognosis.^17^ However, for TNBCs that do not show pCR, the likelihood of relapse is high. As additional effective therapies are not currently available, relapse carries a poor prognosis for patients with TNBC.^15^,^17^ We demonstrate here that curcumin induces DNA damage and apoptosis of triple negative breast cancer cells, but not of the non-transformed mammary epithelial cell line MCF12A. In addition, curcumin promotes phosphorylation, total expression, and cytoplasmic retention of the BRCA1 protein. These results suggest that curcumin activates a DNA damage response in TNBC cells, leading to apoptosis, possibly in part because BRCA1 is retained in the cytoplasm where it cannot repair DNA damage.

## Materials and Methods

### Materials

Curcumin (EMD; Gibbstown, NJ) was dissolved in ethanol at a stock concentration of 8 mM. IKK inhibitor wedelolactone (EMD) was dissolved in DMSO at 15 mM stock concentration.

### Cell culture

Triple negative MDA-MB-468 (MDA468), HCC1937, and HCC1806 breast cancer lines, HER2-over-expressing SKBR3 cells, ER-alpha-positive MCF7, and non-transformed mammary epithelial line MCF12A were purchased from ATCC (Manassas, VA). HCC1806 cells were maintained in RPMI with 5% fetal calf serum (FCS); MCF12A cells were maintained in DMEM/F12 supplemented with 5% horse serum, 20 ng/mL EGF, 10 μg/mL insulin, and 0.5 μg/mL hydrocortisone; all other lines were maintained in Dulbecco’s modified Eagle’s medium (DMEM) supplemented with 10% FCS; all cell lines were maintained on 1% penicillin/streptomycin and incubated at 37 °C with 5% CO_2_ in a humidified incubator.

### Dose-response assays

Cells were treated with two-fold serial dilutions of curcumin for 72 hours (h), at which point cell survival was measured by trypan blue exclusion. Control cultures were treated with ethanol corresponding to the highest dose of curcumin, since curcumin is dissolved in ethanol. In addition, ethanol was added to lower dose curcumin treatment groups to make up the difference in volume of ethanol between the highest curcumin treatment group and the lower dose groups. Experiments were done at least in duplicate and performed at least twice. Cell viability is expressed as a percentage of control ethanol-treated cells per individual cell line; error bars represent standard deviation between replicates.

### Immunoblotting

Cells were lysed in RIPA buffer (Cell Signaling; Danvers, MA), which includes 0.1% NP40, supplemented with protease and phosphatase inhibitor cocktails (Sigma; St. Louis, MO). Total protein extracts (50 μg) were run on SDS-PAGE and immunoblotted using the following antibodies overnight at the indicated dilutions: from EMD, HER2 (erbb2/neu) monoclonal 3B5 used at 1:1000; from Cell Signaling, p-p65 NF-kB monoclonal (93H1) used at 1:250, total p65 NF-kB monoclonal (C22B4) used at 1:250, PARP polyclonal used at 1:200; from Bethyl Labs (Montgomery, TX), p-S1189 and p-S1280 BRCA1 polyclonals used at 1:200 each; from Santa Cruz Biotechnology (Santa Cruz, CA), BRCA1 (C-20) and p-S988 BRCA1 polyclonals used at 1:200 each, ER alpha (G-20), survivin monoclonal D-8 used at 1:500; and from Sigma, β-actin monoclonal used at 1:20,000. Secondary antibodies were chosen according to the species of origin of the primary antibody. Protein bands were detected using the Odyssey Imaging System (Li-Cor Biosciences; Lincoln, NB).

### Anchorage-independent growth assays

Cells were plated in duplicate at 15 × 10^5^ in 6-well dishes in 1ml of matrigel (BD Biosciences; San Jose, CA) diluted 3:1 (media:matrigel). The matrigel cell suspension was allowed to solidify for at least 2 h at 37 °C. Once the matrigel solidified, 2 ml of media containing the ethanol control or curcumin (5 or 15 uM) was added to each well. The cells were maintained for approximately 2 weeks, during which media containing either control or curcumin was replenished twice a week. Photographs were taken with an Olympus IX50 inverted microscope at 4X magnification. To quantify the cell number, matrigel was digested using dispase (BD Biosciences). Briefly, the media was removed from each well and 2 mL of dispase was added per well and incubated at 37 °C for 1 h. Each 3 mL sample was then transferred to a centrifuge tube and 10 mM EDTA was added to stop the enzymatic activity of dispase. Each sample was centrifuged at 1000 rpm for 5 minutes (min) and washed 3 times with phosphate-buffered saline (PBS). Cells were then counted by trypan blue exclusion.

### Migration assays

Monolayers of 750,000 HCC1806 cells were plated and grown in a 6-well cell culture dish. Using a p200 pipette tip, confluent cell cultures were scratched down the center. After scratching, the cells were treated with ethanol or 15 μM curcumin. Cell cultures were photographed (Olympus IX50 microscope, 4X magnification) at 0 and 24 h time points.

### Immunofluorescence

Cells (100,000) were plated per chamber in 4-well chamber slides in 500 μl media and incubated overnight. The next day cells were treated with ethanol or curcumin for 24 h. After washing in PBS, cells were fixed with 4% paraformaldehyde and 0.2% gluteraldehyde for 20 min at room temperature with gentle shaking. After washing, cells were permeabilized with 0.5% Triton X for 10 min at room temperature with gentle shaking. Cells were washed with PBS followed by blocking for 15 min with 5% normal goat serum at room temperature. After washing, cells were incubated overnight with the primary antibody (1:100) in 5% NGS in PBS at 4 °C. The next day cells were washed 6 times, 5 min each time with PBS and incubated for 1 hour at room temperature in a light-protected container with secondary antibody (1:250 in 5% NGS) that was chosen according to the species of origin of the primary antibody. The cells were washed 6 times for 5 min with PBS. The chamber was removed and one drop of the mounting medium which contains DAPI (Vectashield, Vector Laboratories, Inc. Burlingame, CA) was added then sealed with a coverslip. The slide was dried for 2 h in the dark at room temperature. Slides are stored at 4 °C. Photographs were taken using Zeiss Axioplan 2 Upright Microscope. Primary antibodies were BRCA1 (C-20) and phospho-gamma H2Ax polyclonal (both from Santa Cruz).

### NF-kB transcription factor activity assay

HCC1806 cells were plated in 100-mm dishes and treated with ethanol or 10 μM curcumin for 6 h or 24 h. After incubation, the cells were lysed for nuclear proteins with Nuclear Extraction Kit per manufacturer protocol (Cayman Chemical, Ann Arbor, MI). Nuclear extracts were used to detect DNA binding activity of NF-kB by using NF-kB (p65) Transcription Factor Assay Kit (Cayman Chemical). Briefly, 10 μl of the nuclear extract was added to the wells coated with a consensus dsDNA sequence in duplicates together with 90 ul complete transcription factor buffer and incubated overnight at 4 °C. Blank wells, positive control (TNF alpha-stimulated HeLa cell nuclear extract provided with kit), and non-specific binding (provided with kit) samples were also included on the plate. The next day the wells were washed 5 times and incubated with the NF-kB primary antibody (except the blank wells) for 1 h at room temperature. Wells were washed and incubated with HRP-conjugated secondary antibody (except the blank wells) for 1 h at room temperature. After washing, 100 μl of developing solution was added and incubated for 45 min at room temperature with gentle agitation followed by addition of 100 μl stop solution to each well. The absorbance was read at 450 nm. The reading for nonspecific binding was subtracted from each treatment, and the results were normalized to the nuclear extract concentration. Fold change of each sample relative to the average of untreated samples was determined.

### Statistical analysis

Results were analyzed using a two-tailed Student’s *t* test to assess statistical significance. Values of p < 0.05 were considered statistically significant.

## Results

### Curcumin induces BRCA1 protein expression and phosphorylation in triple negative breast cancer cells

**Figure 1.**
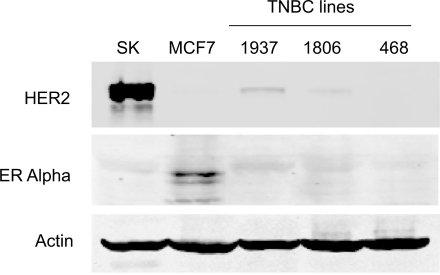
**Estrogen receptor alpha and HER2 expression status of breast cancer cell lines.** SKBR3, MCF7, HCC1937, HCC1806, and MDA468 cell lysates (50 μg) were immunoblotted for ER alpha, HER2, and actin. In comparison to ER+ MCF7 cells and HER2-over-expressing SKBR3 cells, HCC1937, HCC1806, and MDA468 are ER-negative and do not over-express HER2.

ER-alpha and HER2 expression status of HCC1937, HCC1806, and MDA468 cell lines was assessed by immunoblot, using MCF7 cells as a positive control for ER-alpha and SKBR3 cells as a positive control for HER2 (Fig. 1). Relative to SKBR3 cells, which harbor amplification of the *her2* gene with subsequent over-expression of the HER2 protein, HCC1937, HCC1806, and MDA468 express low levels of HER2. In addition, these three lines do not show expression of ER-alpha, similar to SKBR3 which are known to be ER-alpha-negative, and compared to MCF7 cells, which are ER-alpha-positive. PR expression was previously reported as being negative in each of these lines, and other reports have confirmed the ER-negative and low HER2 expression status of these lines.^26^–^29^ Thus, HCC1937, HCC1806, and MDA468 cells represent *in vitro* models of triple negative breast cancer.

**Figure 2.**
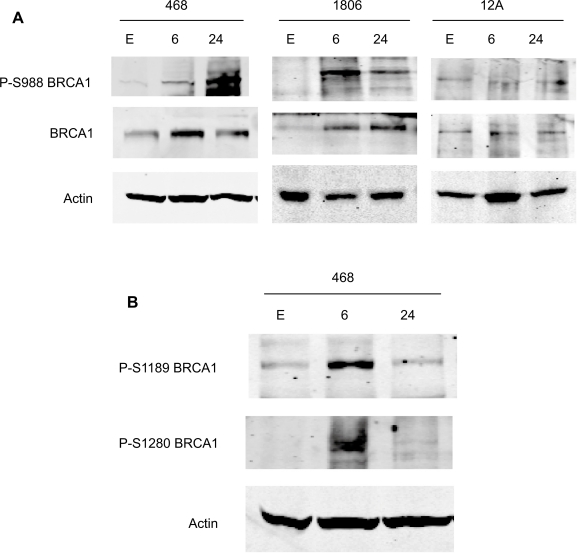
**Curcumin induces BRCA1 expression and phosphorylation. (A)** MDA468, HCC1806, and MCF12A cells were treated with curcumin (10 μM) for 6 or 24 h. As a control, cells were treated for 24 h with the volume of ethanol, *E*, equivalent to that present in 10 μM curcumin. Total cell lysates were immunoblotted (50 μg) for total BRCA1 and BRCA1 phosphorylated at serine 988 (p-S988), a chk2-specific phosphorylation site. Curcumin induced S988 phosphorylation and increased total levels of BRCA1 within 6 h in MDA468 and HCC1806 triple negative cells, but not in MCF12A non-transformed cells. **(B)** Immunoblots for phosphorylated serine 1189 and serine 1280 (ATM phosphorylation sites) were performed on lysates (50 μg) from MDA468 cells treated with curcumin (10 μM) for 6 or 24 h or ethanol, *E*, for 24 h. Curcumin induced transient phosphorylation of both residues by 6 h, suggesting short-term activation of ATM by curcumin.

BRCA1 dysfunction is often observed in TNBC. BRCA1 function is regulated in part by phosphorylation and in part by cellular localization. Since other dietary polyphenols including resveratrol and indole-3-carbinol have been shown to induce expression of the BRCA1 protein in breast cancer cells,^30^,^31^ we examined the effect of curcumin on BRCA1 in TNBCs. Total BRCA1 protein expression was induced in MDA468 and HCC1806 cells within 6 h of treatment with 10 μM curcumin (Fig. 2A). Phosphorylation at serine 988, a chk2 kinase-specific phosphorylation site on BRCA1,^32^ was also increased in MDA468 and HCC1806 cells within 6 h of curcumin exposure. In contrast, curcumin did not alter expression or phosphorylation of BRCA1 in MCF12A non-transformed mammary epithelial cells. We examined two additional phosphorylation sites using MDA468 as a model of TNBC. Serine 1189 and serine 1280 are ATM kinase phosphorylation sites on BRCA1.^32^ Curcumin induced a transient increase in phosphorylation at both sites, occurring within 6 h and back to baseline again by 24 h (Fig. 2B).

### BRCA1 modulation appears to be independent of curcumin-mediated NF-kB inhibition

**Figure 3.**
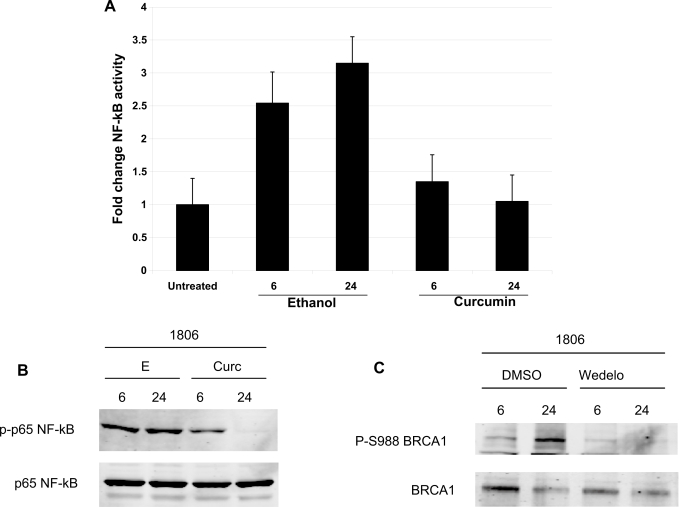
**Modulation of BRCA1 in response to curcumin appears to be independent of NF-kB inhibition.** (**A**) HCC1806 cells were untreated, treated with 10 μM curcumin or corresponding volume of ethanol for 6 h (6) or 24 h (24), and then lysed for nuclear protein. DNA binding activity of NF-kB was determined for nuclear extracts using NF-kB (p65) Transcription Factor Assay colorimetric kit (Cayman Chemical). Samples were run in duplicate. Fold change of the average of each sample relative to the average of untreated samples was determined; error bars reflect standard deviation between duplicates. Curcumin inhibited NF-kB transcription factor activity in HCC1806 cells. (**B**) HCC1806 cells were treated with 10 μM curcumin (*Curc*) or corresponding volume of ethanol (*E*) for 6 h or 24 h. Total protein lysates (50 μg) were immunoblotted for phosphorylated and total p65 NF-kB. Curcumin inhibited phosphorylation of p65 NF-kB consistent with inhibition of NF-kB function. (**C**) HCC1806 cells were treated with 10 μM of IKK inhibitor wedelolactone (*Wedelo*) or corresponding volume of solvent DMSO for 6 h or 24 h. Total protein lysates (50 μg) were immunoblotted for phosphorylated and total BRCA1. In contrast to curcumin, wedelolactone did not induce phosphorylation of S988 on BRCA1 or expression of total BRCA1, suggesting that modulation of BRCA1 may occur independently of IKK inhibition.

Many of the molecular effects of curcumin have been attributed to its ability to potently inhibit the NF-kB transcription factor. We confirmed that curcumin inhibits NF-kB in triple negative breast cancer cells using the HCC1806 line as a model. Curcumin inhibited NF-kB transcription factor function (Fig. 3A) and phosphorylation of p65 NF-kB (Fig. 3B). Next, we compared an IKK inhibitor (wedelolactone) to curcumin for effects on BRCA1 phosphorylation and expression to determine whether curcumin-mediated changes in BRCA1 expression and phosphorylation may be due to IKK inhibition. IKK is an upstream kinase of NF-kB that inhibits NF-kB function; curcumin is thought to inhibit NF-kB via IKK activation. Wedelolactone did not induce phosphorylation or total expression of BRCA1 in contrast to curcumin (Fig. 3C). Thus, since the IKK inhibitor wedelolactone did not induce modulation of the BRCA1 protein, curcumin-mediated changes in BRCA1 may be independent of IKK inhibition.

### Curcumin induces DNA damage and cytoplasmic localization of BRCA1 in triple negative cells with wild-type *brca*1

**Figure 4.**
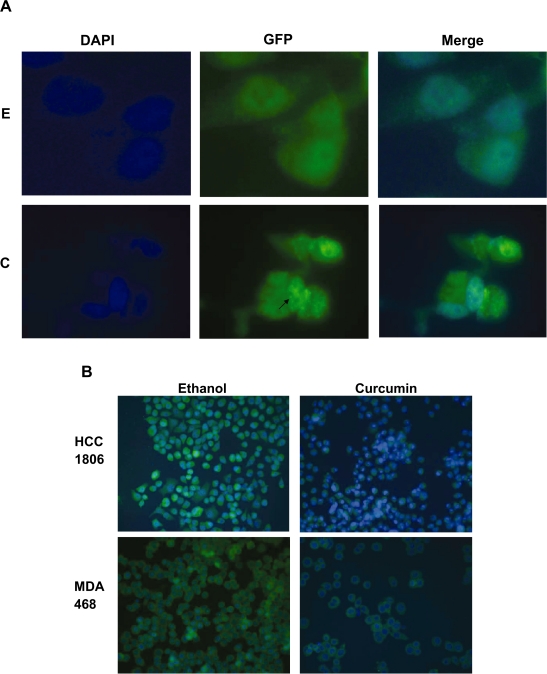

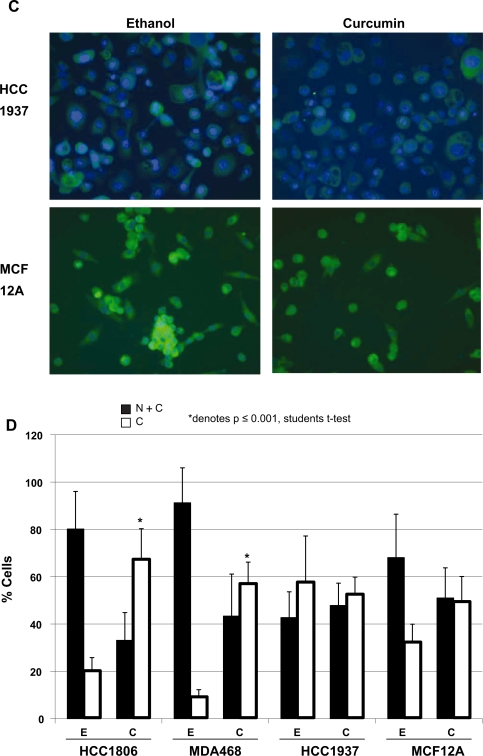
**Curcumin induces DNA damage and cytoplasmic localization of BRCA1.** (**A**) MDA468 cells were treated with ethanol, *E*, or curcumin, *C* (10 μM), for 24 h. Cells were fixed, stained with phospho-gamma H2Ax antibody followed by secondary antibody conjugated to green fluorescent protein (GFP). Phospho-gamma H2Ax foci (arrow) and DAPI nuclear staining were detected by immunofluorescent microscope. Representative DAPI, GFP (phospho-gamma H2Ax), and merged DAPI plus GFP photographs are shown using 100X objective lens. (**B**) HCC1806 and MDA468, and (**C**) HCC1937 and MCF12A cells were treated with ethanol or curcumin (10 μM) for 24 h. Cells were fixed, stained with anti-BRCA1 mouse antibody, followed by secondary GFP-conjugated anti-mouse antibody. BRCA1 localization was detected by immunofluorescent microscope. Representative photographs are shown using 4X objective lens. (**D**) Cells from (B) and (C) above that were in the ethanol, *E*, and curcumin, *C*, treatment groups were counted in five random non-overlapping fields for nuclear + cytoplasmic, *N* + *C*, versus cytoplasmic, *C*, staining only. Error bars represent standard deviation between the five fields per treatment group per line. Curcumin increased BRCA1 localization in the cytoplasm in HCC1806 and MDA468 cells (p ≤ 0.001), but did not show statistically significant changes in localization in HCC1937 and MCF12A cells.

Phosphorylation of ATM/chk2-specific sites on BRCA1 suggests that curcumin may be inducing DNA damage in TNBC cells. To test this hypothesis, we examined phosphorylation of serine 139 on histone H2Ax (gamma-H2Ax), which occurs in response to DNA double-strand breaks. Immunofluorescence showed gamma-H2Ax (GFP) foci in curcumin-treated MDA468 cells but not in control ethanol-treated cells (Fig. 4A). Collectively, these results indicate that curcumin induces DNA damage in triple negative breast cancer cells, which is associated with phosphorylation and expression of the BRCA1 DNA repair protein.

Nuclear localization of BRCA1 is necessary for activation of its transcription factor and DNA repair activity. In response to a DNA damaging agent, cytoplasmic retention of BRCA1 may occur, preventing DNA repair and promoting apoptosis. Immunofluorescence showed that BRCA1 is expressed in both the cytoplasm and nucleus in 80%–100% of control (ethanol-treated) HCC1806 and MDA468 cells (Figs. 4B and 4D). However, in the presence of curcumin (10 μM), the percentage of cells showing only cytoplasmic localization of BRCA1 is increased by 3-fold for MDA468 cells and 6-fold for HCC1806 cells. Both lines showed at least 60% of cells with cytoplasmic sequestration of BRCA1 protein upon treatment with curcumin, a statistically significant (p ≤ 0.001) change versus the control cells (Fig. 4D). On the other hand, 60% of control HCC1937 cells showed BRCA1 localized to the cytoplasm, and curcumin exposure did not change that percentage (Figs. 4C and 4D). Similarly, MCF12A cells did not show statistically significant changes in cytoplasmic localization of BRCA1 in response to curcumin. Thus, BRCA1 localization is shifted to the cytoplasm upon curcumin treatment in the triple negative cell lines MDA468 and HCC1806, which have wild-type *brca*1, but not in the mutant *brca*1 cell line HCC1937, nor in non-transformed mammary epithelial MCF12A cells. These results indicate that curcumin promotes cytoplasmic retention of BRCA1 in triple negative breast cancer cells that have functional BRCA1.

### Curcumin promotes cell death and inhibits anchorage-independent growth and migration of triple negative breast cancer cells

**Figure 5.**
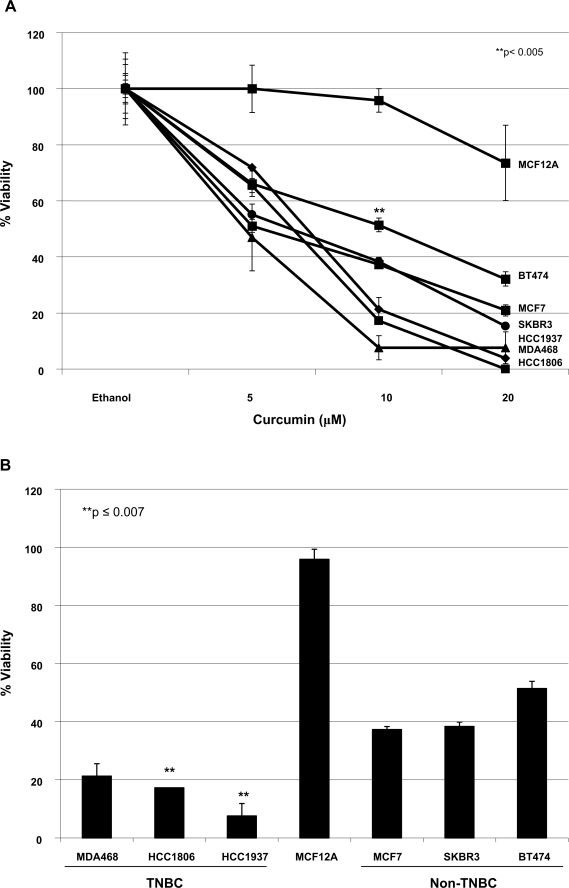

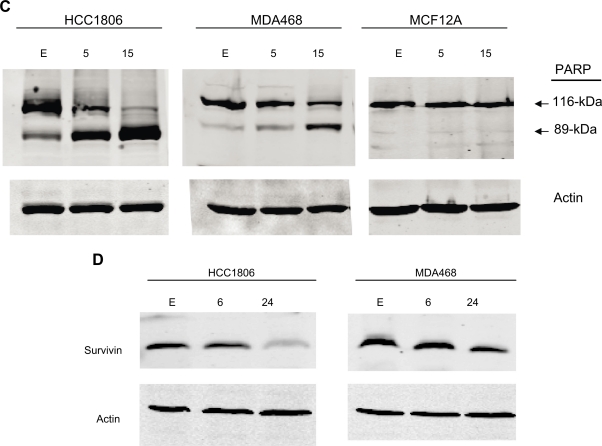
**Curcumin promotes cell death of triple negative breast cancers.** (**A**) Triple negative MDA468, HCC1806, and HCC1937, and ER+/HER2-over-expressing BT474, HER2-over-expressing SKBR3, ER+ MCF7, and non-transformed MCF12A cells were treated with 5, 10, or 20 μM curcumin for 72 h. Control cells were treated with ethanol corresponding to the highest dose of curcumin, since curcumin is dissolved in ethanol. Surviving cells were counted by trypan blue exclusion. For each treatment group, cell viability is shown as a percentage of the ethanol control group per line. Experiments were done in duplicate or triplicate at least twice. Error bars represent standard deviation between replicates. In comparison to MCF12A non-transformed mammary epithelial cells, curcumin inhibited viability of all cancer lines (^**^p < 0.005 at 10 μM and 20 μM). (**B**) Results from the experiment in (**A**) are shown for the 10 μM curcumin dose for comparison of triple negative breast cancer (TNBC) and non-TNBC cells. TNBC cells HCC1806 and HCC1937 showed statistically significant (^**^p < 0.007) higher sensitivity to curcumin versus non-TNBC lines. MDA468 cells showed a trend of being slightly more sensitive to curcumin than non-TNBC cells, although this difference was not statistically significant (p = 0.16, p = 0.14, p = 0.06, respectively for MDA468 versus MCF7, SKBR3, BT474). (**C**) Cells were treated with ethanol, *E*, corresponding to highest dose of curcumin, 5 μM curcumin, or 15 μM curcumin for 24 h. Total lysates (50 μg) were immunoblotted for PARP and actin. HCC1806 and MDA468 showed significant cleavage of PARP consistent with induction of apoptosis within 24 h of curcumin treatment. MCF12A cells did not show evidence of PARP cleavage in response to curcumin, consistent with trypan blue results in (A) which demonstrate that MCF12A non-transformed mammary epithelial cells are not sensitive to curcumin at these doses and time points. (**D**) HCC1806 and MDA468 cells were treated with ethanol, *E*, as a control or 10 μM curcumin for 6 or 24 h. Total lysates (50 μg) were immunoblotted for survivin and actin. Curcumin suppressed expression of the anti-apoptotic protein survivin within 24 h, consistent with induction of apoptosis.

Cytoplasmic retention of DNA repair protein BRCA1 suggests that TNBC cells MDA468 and HCC1806 may be unable to repair curcumin-mediated DNA damage and may undergo apoptosis in response to curcumin exposure. In addition, lack of functional BRCA1 suggests that HCC1937 cells may have an impaired ability to repair DNA damage, and may undergo apoptosis upon treatment with a DNA damaging agent. We examined the biological response of TNBC cell lines to curcumin using trypan blue exclusion viability assays (Fig. 5A). Curcumin induced dose-dependent cell death within 72 h of treatment in all three TNBC lines. In contrast to TNBC cells, the non-transformed mammary epithelial line MCF12A did not display dose-dependent cell death in response to curcumin. Statistical analysis showed that each of the three TNBC lines showed significantly higher response to curcumin at all doses examined (p < 0.005 at 10 μM, p < 0.05 at 5 μM and 20 μM) in comparison to MCF12A cells. Non-TNBC lines MCF7 (ER-positive), SKBR3 (HER2-positive), and BT474 (ER-positive and HER2-positive) also respond to curcumin in a dose-dependent manner. However, the TNBC lines HCC1806 and HCC1937 showed statistically significant (p ≤ 0.007) higher responses to 10 μM curcumin in comparison to non-TNBC lines (Fig. 5B). MDA468 cells showed a trend toward being more sensitive than MCF7, SKBR3, and BT474, but this was not statistically significant (p = 0.16, p = 0.14, p = 0.06, respectively). These dose-response profiles indicate that curcumin induces cell death in breast cancer cells, with triple negative breast cancer cells showing a trend toward increased sensitivity versus ER-positive/HER2-over-expressing cells.

To further examine if curcumin induces apoptosis of TNBC cells, cleavage of poly (ADP-ribose) polymerase (PARP) (Fig. 5C) and expression of anti-apoptotic protein survivin (Fig. 5D) in response to curcumin were measured in MDA468 and HCC1806 cells by immunoblotting. During apoptosis, the full-length 116-kDa PARP protein is cleaved in a caspase-dependent manner into an 89-kDa fragment. Curcumin induced cleavage of PARP at 5 μM and 15 μM doses in both MDA468 and HCC1806 cells. In contrast, PARP cleavage was not observed in MCF12A cells, consistent with dose-response assays indicating that curcumin does not induce death of non-transformed MCF12A cells. In addition, curcumin (10 μM) reduced expression of survivin in MDA468 and HCC1806 cells within 24 h, consistent with induction of apoptosis.

**Figure 6.**
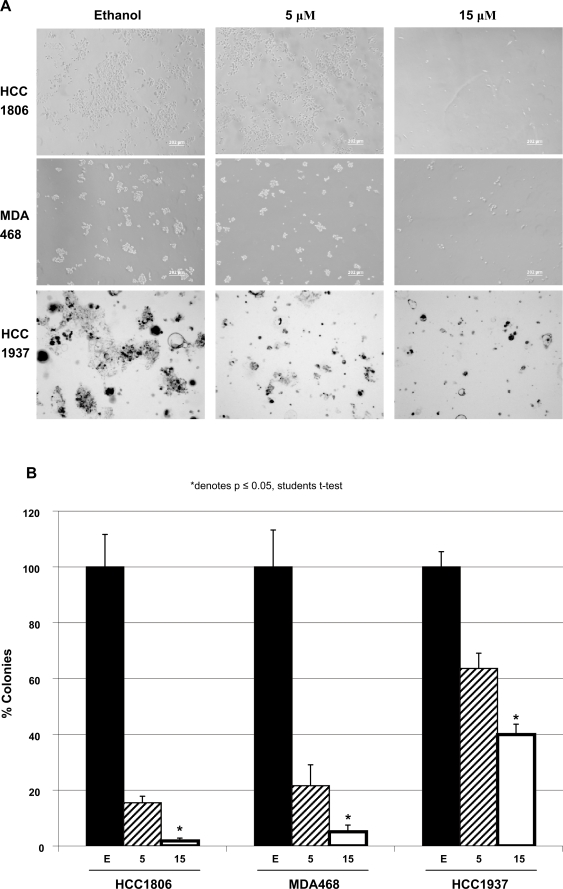

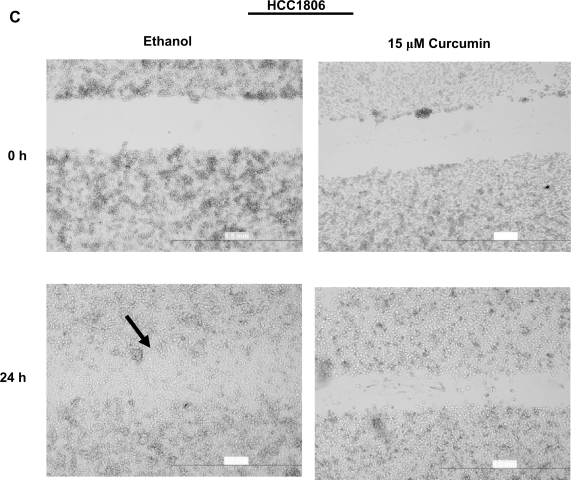
**Curcumin inhibits anchorage-independent growth and migration of triple negative breast cancer cells.** Cells plated in matrigel were treated with ethanol, *E*, corresponding to highest dose of curcumin, 5 μM curcumin, or 15 μM curcumin. Media plus drug (or ethanol) was changed twice a week for two weeks. Experiments were done in duplicate and performed twice. (A) Representative photographs are shown using 4X objective lens with a 202-μm magnification bar shown at the right of photo. (B) For HCC1806 and MDA468 cells, matrigel was dissolved using dispase and cells were counted by trypan blue exclusion. Because of clumping, HCC1937 colonies were counted by microscopic examination. Number of viable colonies is shown as a percentage of the control per line. Error bars represent standard deviation between replicates. Curcumin inhibited anchorage-independent colony growth in a dose-dependent manner in HCC1806, MDA468, and HCC1937 cells, with statistically significant (^*^p ≤ 0.05) inhibition at 15 μM curcumin in all three lines. (C) HCC1806 cells were plated at confluence. The next day cells were scratched down the middle and then treated with ethanol or 15 μM curcumin for 24 h. Representative photos taken with 4X objective lens are shown for 0 h (no curcumin or ethanol treatment) and 24 h. Arrow shows closed wound in control cells, indicating migration of HCC1806 cells within 24 h in presence of ethanol, whereas curcumin prevented migration of HCC1806 TNBC cells.

We next evaluated the effect of curcumin on anchorage-independent growth of TNBCs (Fig. 6A). In comparison to ethanol-treated control cultures, 15 μM curcumin resulted in statistically significant prevention of anchorage-independent growth of all TNBC cell lines (Fig. 6B). To assess the ability of curcumin to prevent migration of TNBCs, we performed in vitro wound healing or “scratch” assays on HCC1806 (Fig. 6C) cells. (MDA468 and HCC1937 cell lines do not migrate well in vitro, and thus were not used for this assay). Curcumin (15 μM) inhibited HCC1806 cell migration in comparison to ethanol-treated control cells, which migrated to close wounds almost completely within 24 h. Thus, curcumin promotes apoptosis and blocks anchorage-independent growth and migration of triple negative breast cancer cells.

## Discussion

The current study demonstrates that curcumin induces DNA damage and apoptosis in triple negative breast cancer cells in association with increased expression, phosphorylation, and cytoplasmic retention of the BRCA1 protein. Phosphorylation occurred on ATM- and chk2-specific sites of BRCA1, consistent with activation of a DNA damage response. In addition, phospho-gamma H2Ax foci were detected in curcumin-treated cells, indicating that the type of DNA lesion produced by curcumin is a double-strand break. Ultimately, although curcumin-mediated DNA damage caused increased expression and phosphorylation of the DNA repair protein BRCA1, the cytoplasmic retention of BRCA1 likely prevents DNA repair from occurring. Thus, cells ultimately undergo apoptosis in response to curcumin.

In contrast to TNBC cells, DNA damage and apoptosis were not observed in curcumin-treated non-transformed mammary epithelial MCF12A cells. These results suggest that curcumin may target cancer cells, with limited non-specific toxicity toward non-cancerous cells. The mutant *brca*1 HCC1937 line did not show increased cytoplasmic retention of BRCA1 in response to curcumin, most likely because BRCA1 is non-functional in these cells and does not need to be shuttled to the cytoplasm in order to prevent its activity. HCC1937 cells did undergo significant apoptosis upon curcumin treatment, however, indicating that mutant *brca*1 TNBC (usually the inherited form) may also benefit from curcumin-based therapy. Since most sporadic TNBCs have down-regulated wild-type *brca*1, similar to HCC1806 cells, a majority of TNBCs may benefit from curcumin-based treatment strategies.

Curcumin has been well-studied as a potential anti-cancer agent for the past decade.^1^ Multiple mechanisms of action including inhibition of NF-kB and STAT3 transcription factor activities have been proposed. Ours is the first study to show that curcumin actually promotes DNA damage with subsequent effects on the BRCA1 DNA repair protein. Interestingly, these results are consistent with other studies showing that indole-3-carbinol (derived from cruciferous vegetables) and resveratrol (derived from red grapes) induce expression of BRCA1.^30^,^31^ Collectively, these studies point toward a trend of diet-derived polyphenol compounds in modulating the BRCA1 protein in cancer cells. Since BRCA1 dysfunction is linked to TNBC, and since our results show apoptotic effects of curcumin in TNBC, a potential role for dietary compounds in prevention or complimentary treatment of TNBC warrants further study.

Our findings also showed a trend of triple negative breast cancer cells being more sensitive to curcumin than non-TNBCs, suggesting a potential new line of therapy for this subset of breast cancers. In addition to inducing cell death, curcumin prevented migration and anchorage-independent growth of TNBCs. Anchorage-independent growth is a hallmark of transformed cells, representative of the fact that cancer cells can proliferate in the absence of cell adhesion,^33^ and *in vitro* migration may predict the metastatic potential of a cancer cell line. Inhibition of anchorage-independent growth and migration are thus considered important pre-clinical support for the potential efficacy of an experimental therapeutic. Based on these results, curcumin has potential anti-cancer efficacy against TNBCs. Curcumin may also benefit non-TNBCs, as shown by our dose-response data; however, the results for TNBCs are particularly exciting, as currently available treatments for TNBCs are extremely limited.

A significant limitation for the clinical use of curcumin is its poor bioavailability.^1^ Several analogues of curcumin have been chemically synthesized and show increased potency and bioavailability relative to the parental compound.^34^,^35^ In addition, strategies for modified delivery of curcumin including polymeric nanoparticle-encapsulated curcumin (“nanocurcumin”), liposomal preparations, and phospholipid complex formulations are being developed and tested for improved bioavailability and potency *in vivo*.^36^,^37^ Based on our results, studies examining curcumin analogues and improved approaches for delivering curcumin to triple negative breast cancers are warranted. These future studies should include *in vivo* xenograft studies of the efficacy of curcumin analogues against TNBCs, which we have not done with the parental compound due to the issue of limited bioavailability. In addition, combination effects of curcumin analogues with currently used chemotherapeutic agents (taxanes, cisplatin, and anthracyclines) in TNBC should be examined.

In summary, we report here that curcumin induces DNA damage and modulates BRCA1 protein expression, phosphorylation, and cellular localization in triple negative breast cancer cells with wild-type *brca*1, but not in a cell line with mutant *brca*1, nor in non-transformed mammary epithelial cells. DNA damage and cytoplasmic retention of BRCA1 post-curcumin treatment are associated with apoptosis of TNBC cells. Treatment options for patients with TNBC who relapse after chemotherapy are currently limited. Identification of novel anti-cancer agents such as curcumin and potentially curcumin analogues could provide a novel, non-toxic therapy for patients with TNBCs, which could lead to improved survival.

## Abbreviations

cdk: cyclin-dependent kinase;
NF-kB: nuclear factor kappa B;
EGFR: epidermal growth factor receptor;
HER2: human epidermal growth factor receptor 2;
ER: estrogen receptor;
PR: progesterone receptor;
TNBC: triple negative breast cancer;
pCR: pathologic complete response;
PARP: poly (ADP-ribose) polymerase;
dsDNA: double-stranded DNA.

## References

[1] Strimpakos AS, Sharma RA (2008). Curcumin: preventive and therapeutic properties in laboratory studies and clinical trials. Antioxid Redox Signal.

[2] Kunnumakkara AB, Guha S, Krishnan S, Diagaradjane P (2007). Curcumin potentiates antitumor activity of gemcitabine in an orthotopic model of pancreatic cancer through suppression of proliferation, angiogenesis, and inhibition of nuclear factor-kappaB-regulated gene products. Cancer Res.

[3] Villegas I, Sánchez-Fidalgo S, Alarcón de la Lastra C (2008). New mechanisms and therapeutic potential of curcumin for colorectal cancer. Mol Nutr Food Res.

[4] Li M, Zhang Z, Hill DL, Wang H, Zhang R (2007). Curcumin, a dietary component, has anticancer, chemosensitization, and radiosensitization effects by down-regulating the MDM2 oncogene through the PI3K/mTOR/ETS2 pathway. Cancer Res.

[5] Barve A, Khor TO, Hao X, Keum YS (2008). Murine prostate cancer inhibition by dietary phytochemicals-curcumin and phenyethylisothiocyanate. Pharm Res.

[6] Narasimhan M, Ammanamanchi S (2008). Curcumin blocks RON tyrosine kinase-mediated invasion of breast carcinoma cells. Cancer Res.

[7] Aggarwal BB, Shishodia S, Takada Y, Banerjee S (2005). Curcumin suppresses the paclitaxel-induced nuclear factor-kappaB pathway in breast cancer cells and inhibits lung metastasis of human breast cancer in nude mice. Clin Cancer Res.

[8] Choudhuri T, Pal S, Agwarwal ML, Das T, Sa G (2002). Curcumin induces apoptosis in human breast cancer cells through p53-dependent Bax induction. FEBS Lett.

[9] Aggarwal BB, Banerjee S, Bharadwaj U, Sung B (2007). Curcumin induces the degradation of cyclin E expression through ubiquitin-dependent pathway and up-regulates cyclin-dependent kinase inhibitors p21 and p27 in multiple human tumor cell lines. Biochem Pharmacol.

[10] Mukhopadhyay A, Banerjee S, Stafford LJ, Xia C (2002). Curcumin-induced suppression of cell proliferation correlates with down-regulation of cyclin D1 expression and CDK4-mediated retinoblastoma protein phosphorylation. Oncogene.

[11] Hong RL, Spohn WH, Hung MC (1999). Curcumin inhibits tyrosine kinase activity of p185neu and also depletes p185neu. Clin Cancer Res.

[12] Squires MS, Hudson EA, Howells L, Sale S (2003). Relevance of mitogen activated protein kinase (MAPK) and phosphotidylinositol-3-kinase/protein kinase B (PI3K/PKB) pathways to induction of apoptosis by curcumin in breast cells. Biochem Pharmacol.

[13] Ramachandran C, Rodriguez S, Ramachandran R, Raveendran Nair PK (2005). Expression profiles of apoptotic genes induced by curcumin in human breast cancer and mammary epithelial cell lines. Anticancer Res.

[14] Schneider BP, Winer EP, Foulkes WD, Garber J (2008). Triple-negative breast cancer: risk factors to potential targets. Clin Cancer Res.

[15] Liedtke C, Mazouni C, Hess KR, André F (2008). Response to neoadjuvant therapy and long-term survival in patients with triple-negative breast cancer. J Clin Oncol.

[16] Harris LN, Broadwater G, Lin NU (2006). Molecular subtypes of breast cancer in relation to paclitaxel response and outcomes in women with metastatic disease: results from CALGB 9342. Breast Cancer Res.

[17] Carey LA, Dees EC, Sawyer L, Gatti L (2007). The triple negative paradox: primary tumor chemosensitivity of breast cancer subtypes. Clin Cancer Res.

[18] Scully R, Chen J, Plug A, Xiao Y (1997). Association of BRCA1 with Rad51 in mitotic and meiotic cells. Cell.

[19] Scully R, Chen J, Ochs RL, Keegan K (1997). Dynamic changes of BRCA1 subnuclear location and phosphorylation state are initiated by DNA damage. Cell.

[20] Zhong Q, Chen CF, Li S, Chen Y (1999). Association of BRCA1 with the hRad50-hMre11-p95 complex and the DNA damage response. Science.

[21] Gudmundsdottir K, Ashworth A (2006). The roles of BRCA1 and BRCA2 and associated proteins in the maintenance of genomic stability. Oncogene.

[22] Melchor L, Honrado E, García MJ, Alvarez S (2008). Distinct genomic aberration patterns are found in familial breast cancer associated with different immunohistochemical subtypes. Oncogene.

[23] Tirkkonen M, Johannsson O, Agnarsson BA, Olsson H (1997). Distinct somatic genetic changes associated with tumor progression in carriers of BRCA1 and BRCA2 germ-line mutations. Cancer Res.

[24] Staff S, Isola J, Tanner M (2003). Haplo-insufficiency of BRCA1 in sporadic breast cancer. Cancer Res.

[25] Murray MM, Mullan PB, Harkin DP (2007). Role played by BRCA1 in transcriptional regulation in response to therapy. Biochem Soc Trans.

[26] Köster F, Engel JB, Schally AV, Hönig A (2008). Triple-negative breast cancers express receptors for growth hormone-releasing hormone (GHRH) and respond to GHRH antagonists with growth inhibition. Breast Cancer Res Treat.

[27] Hegde PS, Rusnak D, Bertaiux M, Alligood K (2007). Delineation of molecular mechanisms of sensitivity to lapatinib in breast cancer cell lines using global gene expression profiles. Mol Cancer Ther.

[28] Han Y, Yang L, Suarez-Saiz F, San-Marina S (2007). Wilms’ tumor 1 suppressor gene mediates antiestrogen resistance via down-regulation of estrogen receptor-alpha expression in breast cancer cells. Mol Cancer Res.

[29] Neve RM, Chin K, Fridlyand J, Yeh J (2006). A collection of breast cancer cell lines for the study of functionally distinct cancer subtypes. Cancer Cell.

[30] Fustier P, Le Corre L, Chalab N, Vissac-Sabatier C (2003). Resveratrol increases BRCA1 and BRCA2 mRNA expression in breast tumour cell lines. Br J. Cancer.

[31] Fan S, Meng Q, Auborn K, Carter T, Rosen EM (2006). BRCA1 and BRCA2 as molecular targets for phytochemicals indole-3-carbinol and genistein in breast and prostate cancer cells. Br J. Cancer.

[32] Ouchi T (2006). BRCA1 phosphorylation: biological consequences. Cancer Biol Ther.

[33] Kang JS, Krauss RS (1996). Ras induces anchorage-independent growth by subverting multiple adhesion-regulated cell cycle events. Mol Cell Biol.

[34] Thomas SL, Zhong D, Zhou W, Malik S (2008). EF24, a novel curcumin analog, disrupts the microtubule cytoskeleton and inhibits HIF-1. Cell Cycle.

[35] Robinson TP, Hubbard RB, Ehlers TJ, Arbiser JL (2005). Synthesis and biological evaluation of aromatic enones related to curcumin. Bioorg Med Chem.

[36] Bisht S, Feldmann G, Soni S, Ravi R, Karikar C, Maitra A (2007). Polymeric nanoparticle-encapsulated curcumin (“nanocurcumin”): a novel strategy for human cancer therapy. J Nanobiotechnology.

[37] Bisht S, Maitra A (2009). Systemic Delivery of Curcumin: 21st Century Solutions for an Ancient Conundrum. Curr Drug Discov Technol.

